# Supplementary material for: Bioinspired bio-voltage memristors
Source: Nat Commun. 2020 Apr 20;11:1861. doi: 10.1038/s41467-020-15759-y (PMC7171104; doi:10.1038/s41467-020-15759-y)
Supplement: Supplementary file 1 — Supplementary Information [file 41467_2020_15759_MOESM1_ESM.pdf]

Supplementary Information

Bioinspired Bio-Voltage Memristors

Tianda Fu[1], Xiaomeng Liu[1], Hongyan Gao[1], Joy E. Ward[2], Xiaorong Liu[3], Bing Yin[1], Zhongrui Wang[1], Ye Zhuo[1], David J. F. Walker,[2] J. Joshua Yang[1], Jianhan Chen[3,4,5], Derek R. Lovley[2,4], Jun Yao[1,4,]

---

1. Department of Electrical and Computer Engineering, University of Massachusetts, Amherst, MA, USA.
2. Department of Microbiology, University of Massachusetts, Amherst, MA, USA.
3. Department of Chemistry, University of Massachusetts, Amherst, MA, USA.
4. Institute for Applied Life Sciences (IALS), University of Massachusetts, Amherst, MA, USA.
5. Department of Biochemistry and Molecular Biology, University of Massachusetts, Amherst, MA, USA.

This PDF file includes:

Supplementary Figures 1 to 23
Supplementary References

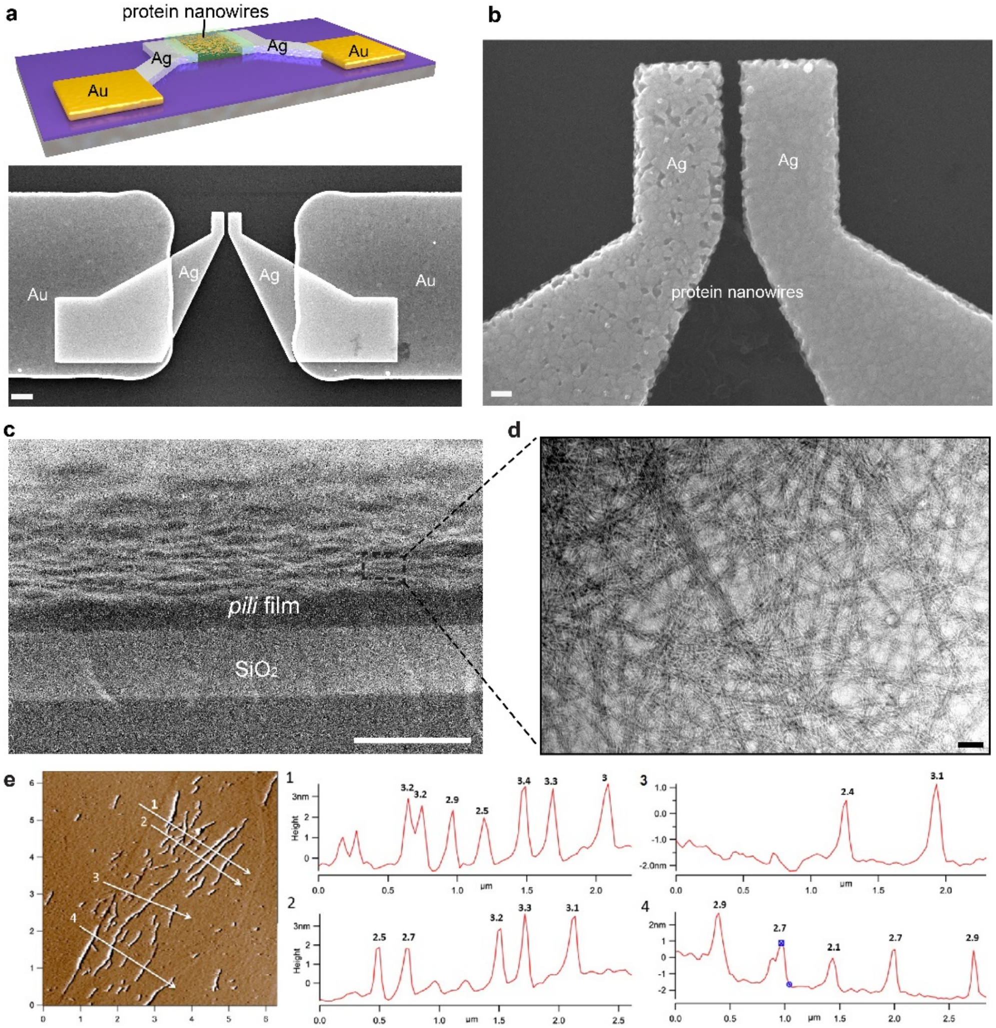

Supplementary Figure 1 | Device fabrication and structure. (**a**) (Top panel) Schematic of a nanowire memristor device, showing a protein-nanowire thin film sandwiched in a nanogap defined by a pair of Ag electrodes that further connect to Au contacts (for probe access). (Bottom panel) Scanning electron microscopy (SEM) image of a typical as-made nanowire device. Scale bar, 1 µm. (**b**) Zoom-in SEM image of the nanogap region in the device. Note that nanowire film was less visible under SEM. Scale bar, 100 nm. (**c**) SEM cross-section view of a nanowire film prepared by using the same dropcasting procedure involved in nanowire-memristor fabrication. Scale bar, 1 µm. (**d**) Transmission electron microscopy (TEM) image of a few nanowire layers. Scale bar, 100 nm. (**e**) Atomic force microscope (AFM) measurement showed a predominance of nanowires with the average diameter of ~2.9±0.35 nm, which was consistent with the reported 3 nm diameter of PilA-based filaments[1] and is inconsistent with the 4 nm diameter of OmcS-based filaments.[2,3]

Note that for TEM imaging, few-layer nanowires were chosen for electron transparency. Consequently, the nanowire density revealed was not a close representation of the nanowire packing density in the actual film (~500 nm thick) used in memristors. As was revealed by cross-section SEM image in (c), the protein nanowires assumed a dense packing configuration in the thin film. The tight nanowire-nanowire interface, revealed by molecular dynamics simulations (Supplementary Figure 2), acted more like the grain boundary in inorganic dielectrics.[1] As a result, the nanowire film behaved like a biological dielectric that allowed for standard lithographic patterning (Supplementary Figure 3), which was completely different from previous loose 'nanowire mesh' used in memristors.[4,5]

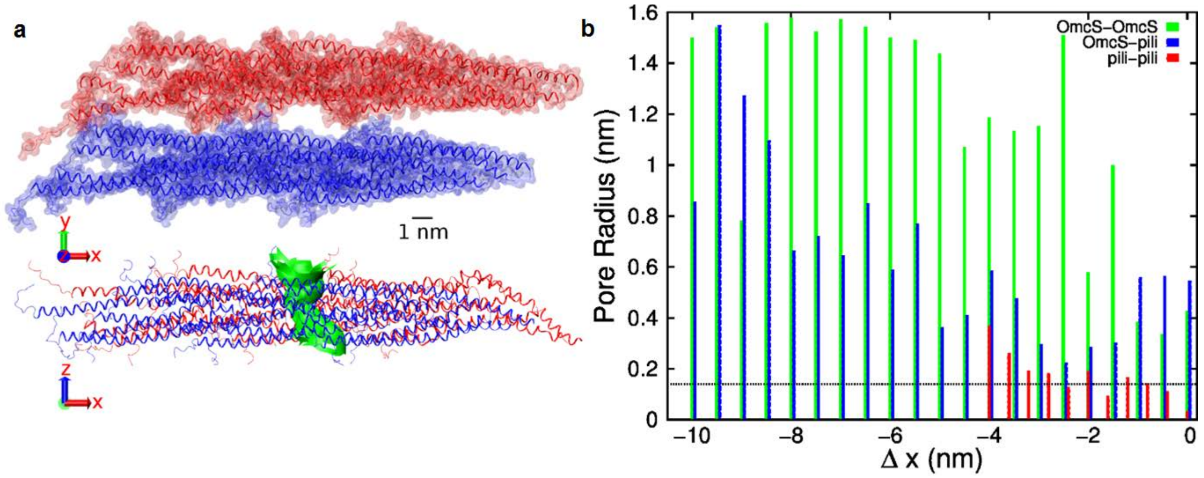

Supplementary Figure 2 | Protein nanowire interfaces. Simulation details are described in the ***Methods***. Recent development has shown the possibility of the coexistence of pili and OmcS nanowire structures,[1-3] although our characterization indicated the predominance of pili nanowires (Supplementary Figure 1e). We modeled different nanowire-nanowire interfaces to provide a comprehensive view. The nanowire-nanowire interface in the simulation involved two nanowire filaments in parallel configurations. (**a**) Representative snapshot of a pili-pili interface and the water-permeable pore (green) from molecular modelling. The blue filament was set as the reference filament, with its principal axis oriented along *x*-axis. The red filament, in parallel with the blue one, was mobile, which could both translate along *y*-axis and rotate along its filament axis. Similar procedure was adopted in the modelling of OmcS-OmcS and OmcS-pili nanowire interfaces. (**b**) Pore radius at each shift distance (Δx) along *x*-axis for the most stable packing conformation in OmcS-OmcS (green), OmcS-pili (blue), and pili-pili nanowire interfaces (red). The black dashed line indicates the radius of water molecule.

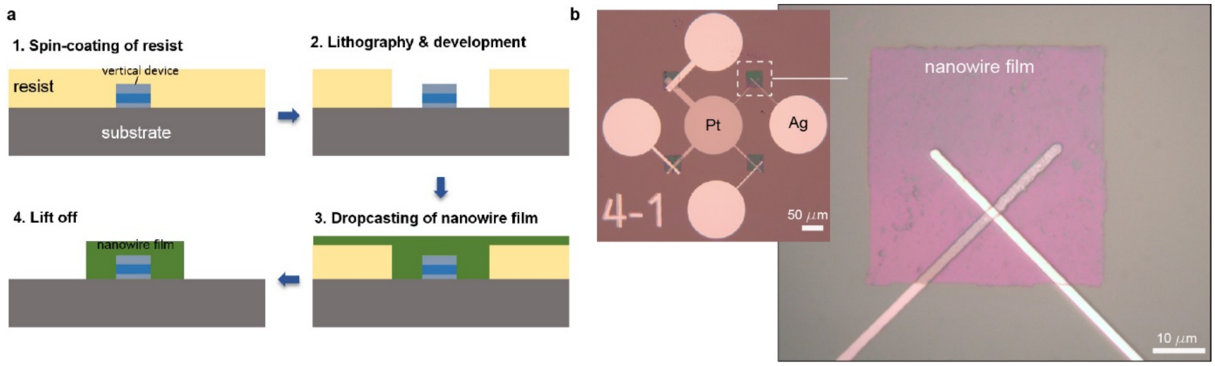

Supplementary Figure 3 | Proof-of-concept demonstration of patterning protein-nanowire film in vertical memristors. (**a**) Schematics of patterning nanowire film by using standard lithography and lift-off processes. Specifically, (1) ~500 nm thick photoresist (Microposit S1805) was spin-coated on the fabricated vertical Ag-SiO_2-Pt devices. (2) The vertical device was then exposed (within a window size ~50×50 μm^2) by standard exposure (with alignment) and development processes. (3) The protein-nanowire solution was dropcasted into the exposed window region and naturally dried in the ambient environment. The thickness (~500 nm) was controlled by dropcasting volume (see Methods). (4). A final life-off in acetone yielded Ag-SiO_2-Pt devices embedded in patterned protein-nanowire film. (**b**) Zoom-in optical image of a vertical Ag-SiO_2-Pt device (size ~2×2 μm^2) embedded in a patterned protein-nanowire film.

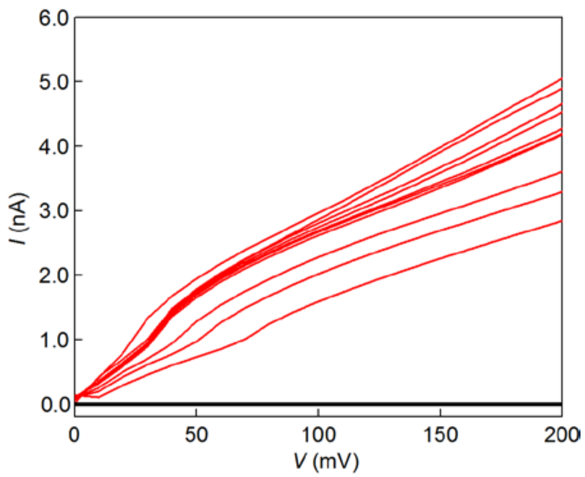

Supplementary Figure 4 | Initial conduction in *protein-nanowire* planar device. Before deposition of nanowire film in the planar Ag-Ag nanogap (~200 nm), the device showed no conduction (black curve). The nanowire film (500 nm thick) was then formed by dropcasting. Low conductance was observed in the nanowire films (N=10, red curves). The estimated conductivity was ~80 μS/cm which was consistent to previous values measured from wild-type protein nanowires prepared in basic solution.[7]

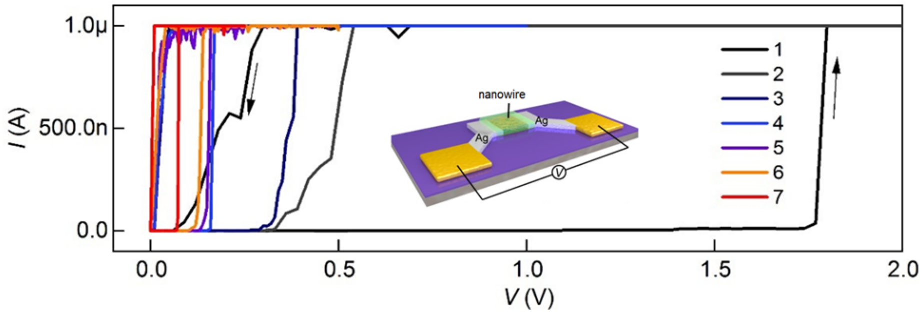

Supplementary Figure 5 | A typical electroforming process in the protein-nanowire memristor. The device was a planar device (inset) with the gap size ~200 nm. The looped voltage sweep was initially up to 2.0 V, during which a sudden conductance jump was observed (black arrow). The voltage in the following sweeps was gradually reduced, within which the sudden conductance jump still maintained with its threshold voltage gradually reducing until stable switching was established (red curve). The numbers indicate the sweep order.

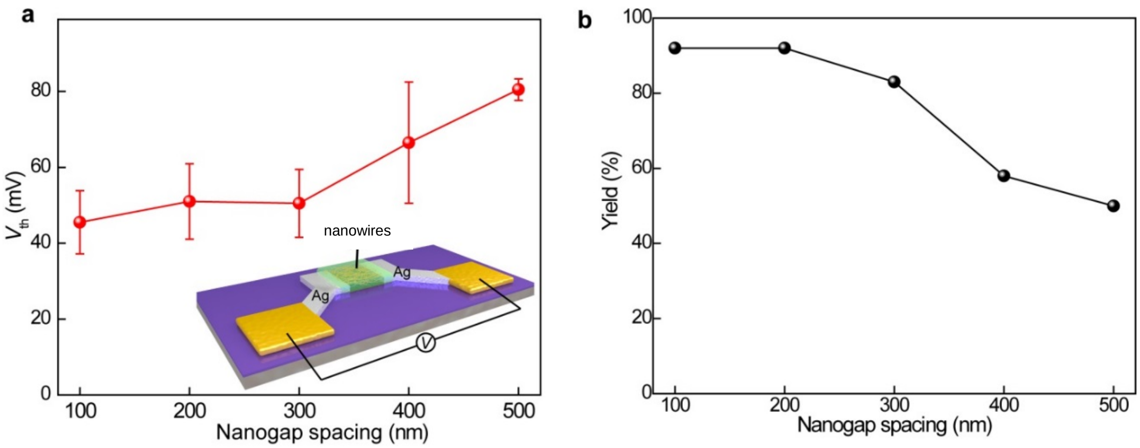

Supplementary Figure 6 | Device statistics. (**a**) The average turn-on threshold voltage (V_{th}) with respect to the nanogap spacing in protein-nanowire memristors. A total number of 60 devices, with 12 for each spacing, were studied. The error bars represent the standard deviation (s.d.). The average V_{th} maintained a value between 40-80 mV. V_{th} is also largely independent of the nanogap size, which is consistent to a filamentary switching mechanism in which the confined switching point generally is independent of the filament length. The inset shows the schematic of the memristive device, with the protein nanowires filled in the nanogap defined by a pair of Ag electrodes. (**b**) Device yield with respect to the nanogap spacing. All the measurements were performed in the ambient environment (RH ~ 40%).

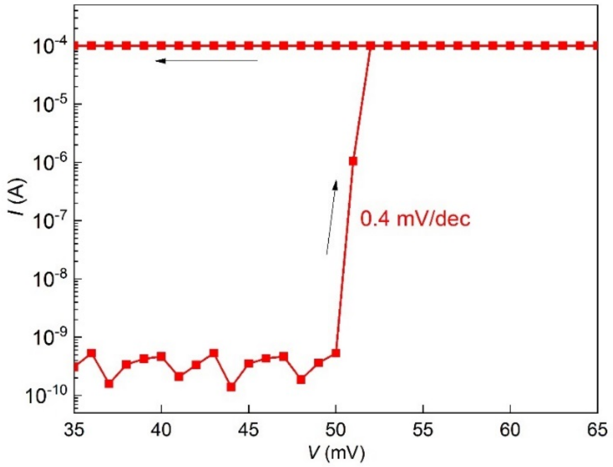

Supplementary Figure 7. Turn-on curve from a typical planar protein-nanowire memristor, showing a steep slope of 0.4 mV/dec.

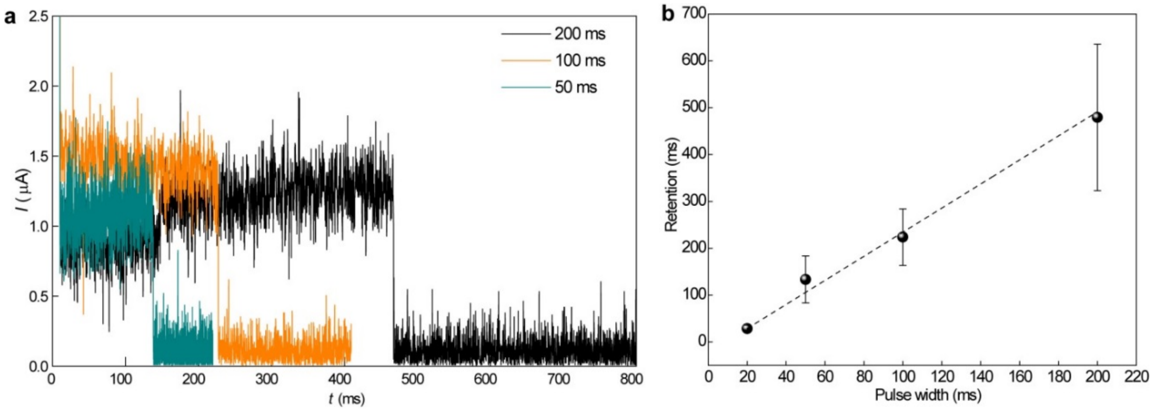

Supplementary Figure 8 | Pulse width-dependent relaxation time in vertical memristors. (**a**) Representative conductance relaxations from a vertical protein-nanowire device switched by programming pulses of different widths. The pulse amplitude was kept at 100 mV. The relaxation current was recorded at 10 mV. (**b**) Average relaxation time with respect to the programming pulse width. The error bars represent the standard deviation (s.d.). The approximately linear increase in relaxation time with respect to pulse width is consistent to other studies.[8] Note that this linear fitting does not pass the origin, because only pulses with width >10 ms can turn on the device (Fig. 2d). The extrapolated 0 ms retention at ~10 ms input pulse width is consistent to the experimental result (Fig. 2d).

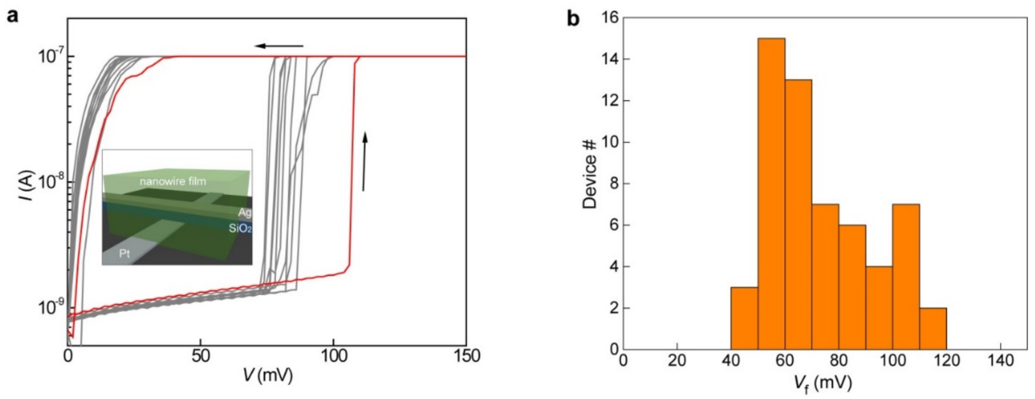

Supplementary Figure 9 | Electroforming in vertical protein-nanowire memristors. (**a**) An electroforming process (red curve) followed by switching *I*-*V*s (gray curves) in a vertical device (size ~ 2×2 μm^2) at RH~35%. (**b**) Statistics of the forming voltages (V_f) from 57 devices, showing an average value of 70±19 mV.

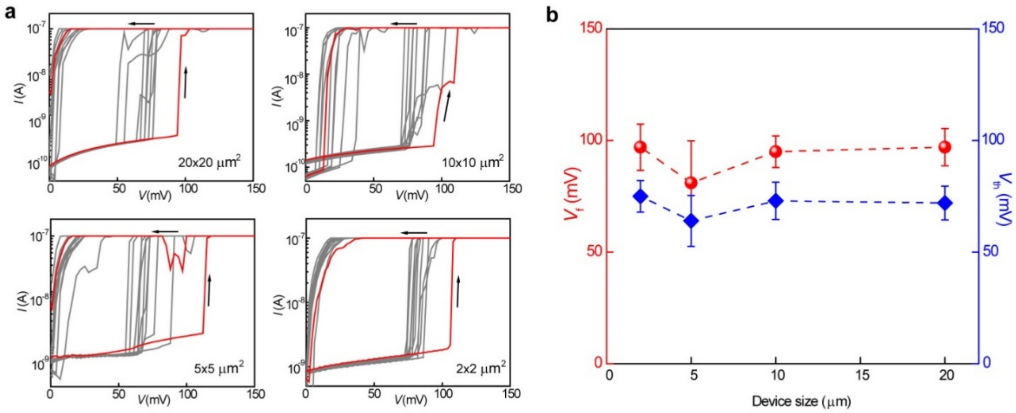

Supplementary Figure 10 | Device scaling in vertical protein-nanowire memristors. (**a**) Representative electroforming process (red curve) followed by 10 consecutive switching *I*-*V*s (gray curves) in vertical devices of different sizes (20×20, 10×10, 5×5, 2×2 μm^2). (**b**) Statistics of the forming voltages (V_f, red dots) and turn-on voltages (V_{th}, blue diamonds) with respect to device sizes (N=5 for each size). The error bars represent the standard deviation (s.d.). The electrical characterizations were performed at RH~35%.

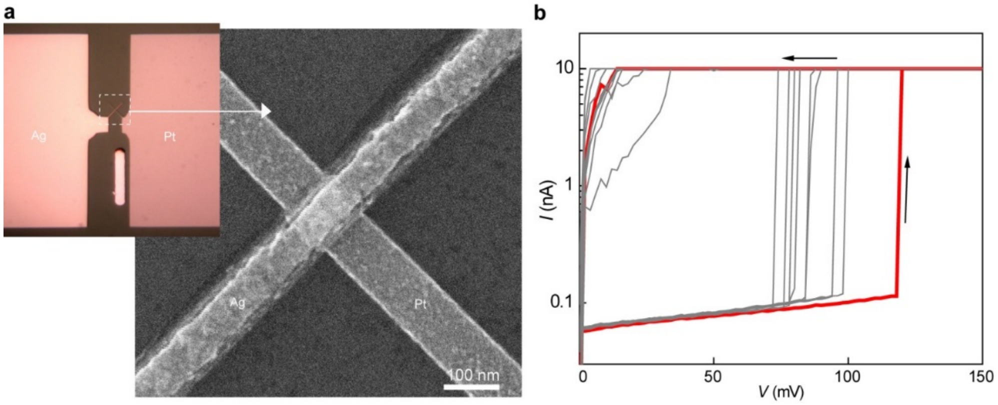

Supplementary Figure 11 | Memristive switching in a 100×100 nm^2 vertical protein-nanowire device. (**a**) Optical and SEM images of the vertical Ag-SiO_2-Pt structure. (**b**) The electroforming process (red curve) followed by switching *I*-*V*s in the device (ambient RH ~35%).

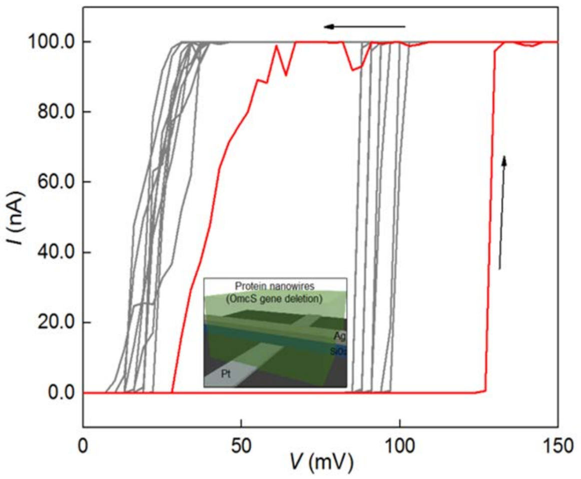

Supplementary Figure 12 | Memristor function with vertical device fabricated with protein nanowires harvested from a strain of *G. sulfurreducens* in which the gene for OmcS was deleted. To further clarify whether OmcS-based nanowire filaments are a contributing factor in observed memristive switching (Fig. 2), protein nanowires were harvested from a strain of *G. sulfurreducens* with the gene for OmcS deleted[6] and used to construct vertical device (inset) similar to that in Fig. 2e. The device showed forming (red curve) and memristive switching (gray curves) similar to ones made with wild type protein nanowires (Supplementary Figure 10), indicating that OmcS is not a contributing factor in observed phenomenon.

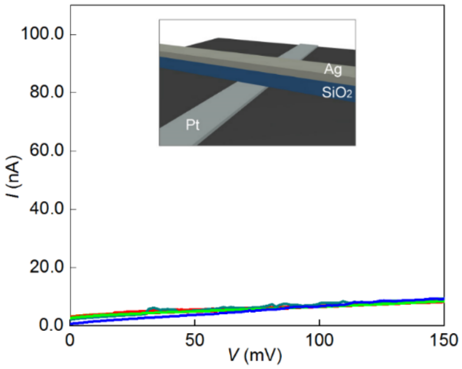

Supplementary Figure 13 | Electroforming in vertical Ag-SiO_2-Pt devices treated with ethanolamine. The ethanolamine buffer involved in nanowire purification was removed by dialyzing the nanowire preparation against deionized water (see Methods). To exclude the possibility that ethanolamine residue could have contributed to the sub-100 mV forming and switching, control vertical Ag-SiO_2-Pt devices (*N*=5) were dropcasted with the same volume of non-diluted 150 mM ethanolamine (without protein nanowires) and dried. None of the devices showed any switching behaviors (color curves) following typical electroforming steps employed in protein-nanowire devices (Supplementary Figure 9).

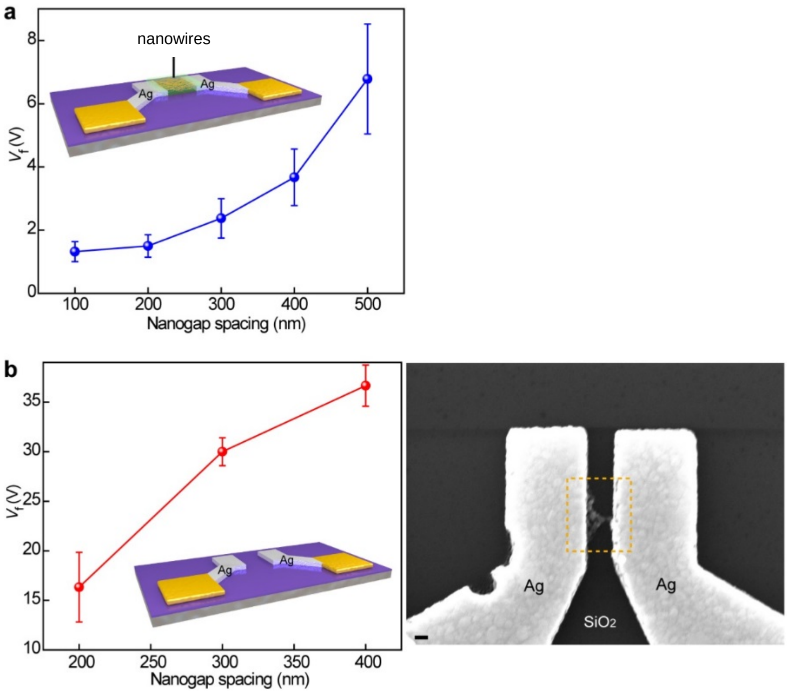

Supplementary Figure 14 | Electroforming in protein-nanowire devices *v.s.* a bare pair of Ag electrodes. (**a**) The average forming voltage (V_f) in protein-nanowire devices with respect to the nanogap spacing. The data were from the same 60 devices studied in Supplementary Figure 6. A general trend of increasing V_f with increasing nanogap spacing was observed, consistent with the field-driven mechanism involved in typical soft breakdown. (**b**) The average forming voltage (V_f) in a bare pair of Ag electrodes (without protein nanowires) with respect to the nanogap spacing. No stable switching was observed at low bias (*e.g.*, <200 mV) afterwards. (Right panel) SEM image showing Ag migration (dashed area) in SiO_2 substrate between a bare pair of Ag electrodes during an electroforming attempt. Scale bar, 100 nm. The error bars in (**a**) and (**b**) represent the standard deviation (s.d.).

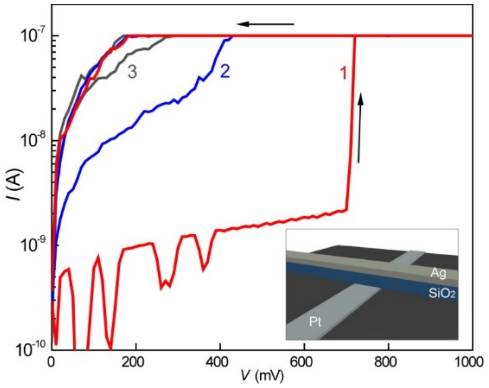

Supplementary Figure 15 | Attempted electroforming and switching in bare Ag-SiO_2-Pt devices. The devices tested (*N*=6) had all the same structure parameters, but did not have protein nanowires (schematic). The testing was carried out in the same ambient condition (RH~35%). The *I*-*V*s above from a representative device shows an initial forming voltage ~700 mV (red curve), but failed to form reliable switching at smaller bias (*e.g.*, <200 mV). The rest 5 devices showed similar forming voltages of ~600, 550, 450, 550, and 600 mV, but also failed to yield stable low-voltage switching.

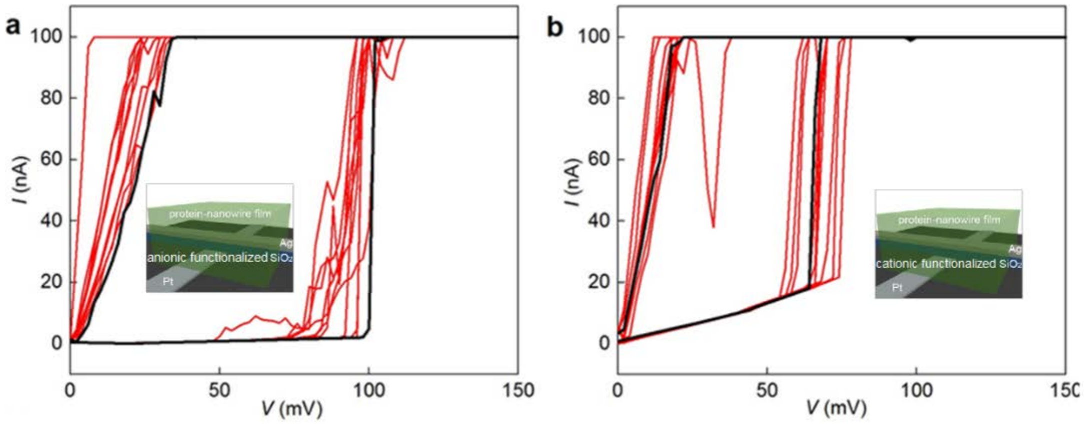

Supplementary Figure 16 | Effect of SiO_2 surface property. Both (**a**) anionic (α-NH2, ω-COOH-terminated polyethylene glycol)) and (**b**) cationic (ω-Amino-terminated poly(ethylene glycol) methyl ether) surfactants were used to change the surface charge states[9,10] and hence surface potential in SiO_2 before depositing protein nanowires. Both devices showed similar bio-voltage switching. The black curves were the initial forming curves. Surface functionalization alone (without protein nanowire) could not yield bio-voltage memristive switching.

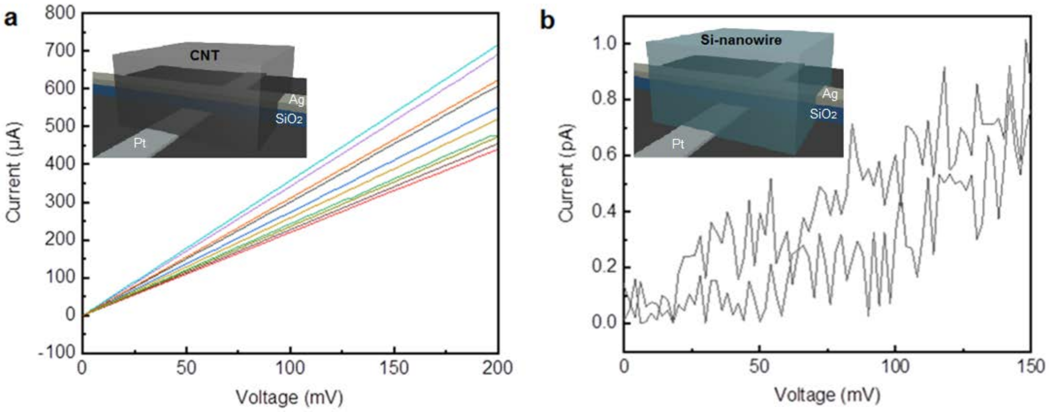

Supplementary Figure 17 | Control nanowire/nanotube devices. The same vertical Ag-SiO_2-Pt memristor structures were embedded in single-walled carbon nanotubes (CNT) and Si-nanowire networks. (**a**) Devices embedded in CNT initially showed high conduction dominated by CNT transport. (**b**) Devices embedded in semiconducting Si-nanowire network showed low conduction (*e.g.*, similar to bare device). None of the devices formed bio-voltage switching following the same forming process. The results are consistent with previous studies,[4,5] in which Ag-based memristors using nanowire mesh/network did not yield low-voltage switching. These 'negative' controls indicate that it is not the percolation material structure that contributes to the effect.

The CNTs (#US4101; US Research Nanomaterials, Inc.), with diameters of 1-2 nm similar to protein nanowire, are expected to yield molecular pores[11] in the film. The thin film was made by dropcasting CNT solution prepared in dimethyl sulfoxide (DMSO). The Si nanowires were grown by a nanocluster-catalyzed vapor–liquid–solid method described previously.[12] Contact printing[13] and multiple transfer processes[14] were used to transfer nanowire films onto the Ag-SiO_2-Pt device.

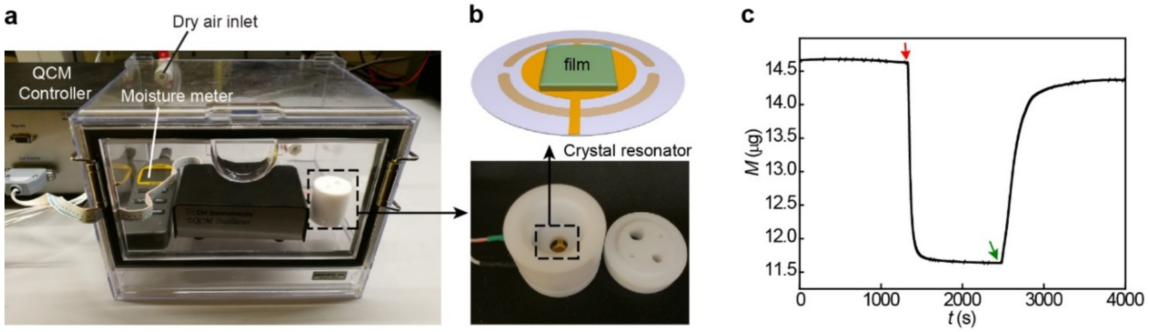

Supplementary Figure 18 | Moisture-adsorption measurement in thin films. (**a**) The measurement setup. It includes a gas-purge desiccator cabinet (H42053-0002; Bel-Art) with an inlet and outlet that allow the controlled flow of dry air, a portable hygrometer (Model 8706; REED Instruments) that can real-time monitor the relative humidity (RH) in the cabinet, and a quartz crystal microbalance (QCM; 400C, CH Instruments) that can monitor the thin-film mass. (**b**) The thin film (*e.g.*, protein nanowires or polyvinylpyrrolidone film) was first deposited on the quartz crystal resonator (top) by dropcasting. The mass sensitivity of the QCM originates from the dependence of the oscillation frequency on the total mass of the metal-coated crystal, including any deposited material. The mass change can be determined by $\Delta m = -\Delta f \cdot A \cdot \frac{\sqrt{\mu\rho}}{2f_0^2}$, where f_0, A, ρ, μ are resonant frequency of crystal's fundamental mode, area of the gold disk on the crystal, crystal's density (2.684 g·cm^{-3}) and shear modulus of quartz (2.947×10^{11} g·cm^{-1}·s^{-2}), respectively.[15] We first determined the mass of the coated thin film (W_{film}) in the ambient environment. Then the resonator was placed in the desiccator cabinet with dry air flowing in to reduce RH to ~0% inside to force out adsorbed moisture in the thin film. During the process, the mass change (ΔW_{film}) that corresponds to the amount of moisture loss in the film, was continuously monitored (reflected by the resonant-frequency change in QCM). (**c**) Weight change in a protein nanowire film measured with QCM. The red arrow indicates the start of moisture removal by dry air, whereas the blue arrow indicates the start of moisture re-adsorption in the protein nanowire film (at ambient RH~35%).

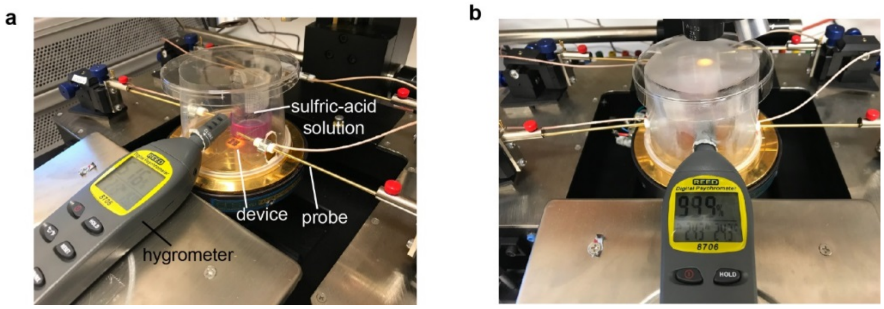

Supplementary Figure 19 | Relative-humidity (RH) control during device measurements. (**a**) The actual setup for controlled RH in device testing environment. The higher RH was controlled by tuning the equilibrium vapor pressure of sulfuric acid solution,[16] *i.e.*, the concentration of sulfuric acid in the open container inside the chamber. (**b**) An example showing the control of RH ~100% in the testing environment.

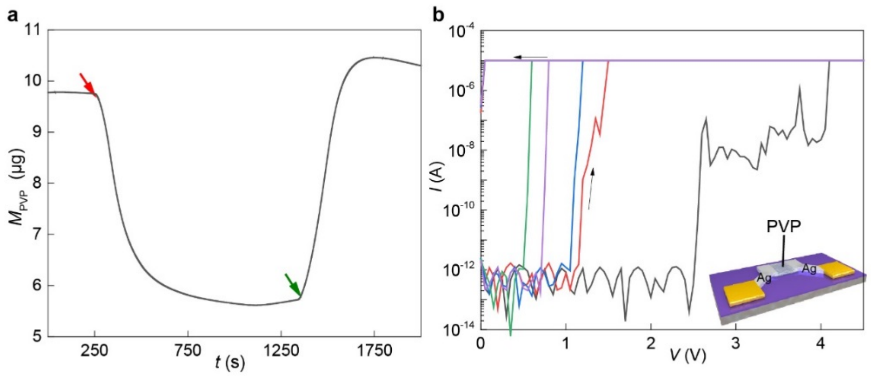

Supplementary Figure 20 | Memristive switching in PVP-Ag device. (**a**) Moisture content measured by QCM (Supplementary Figure 18), which showed that a thin PVP film (~1.8 μm) adsorbed ~40% weigh-percentage moisture. The red arrow indicates when dry air flew in to drive out the moisture, whereas the green arrow indicates the dry-air flow stopped and moisture re-adsorption happened. (**b**) Forming and switching process in a PVP memristor (inset) in the first five consecutive sweeps. The device never went to a turn-on voltage below 0.5 V. The turn-on voltage featured a large variation with the average value >0.5 V. The nanogap spacing between the pair of Ag electrode was 200 nm. The testing was in the ambient environment with RH ~40%.

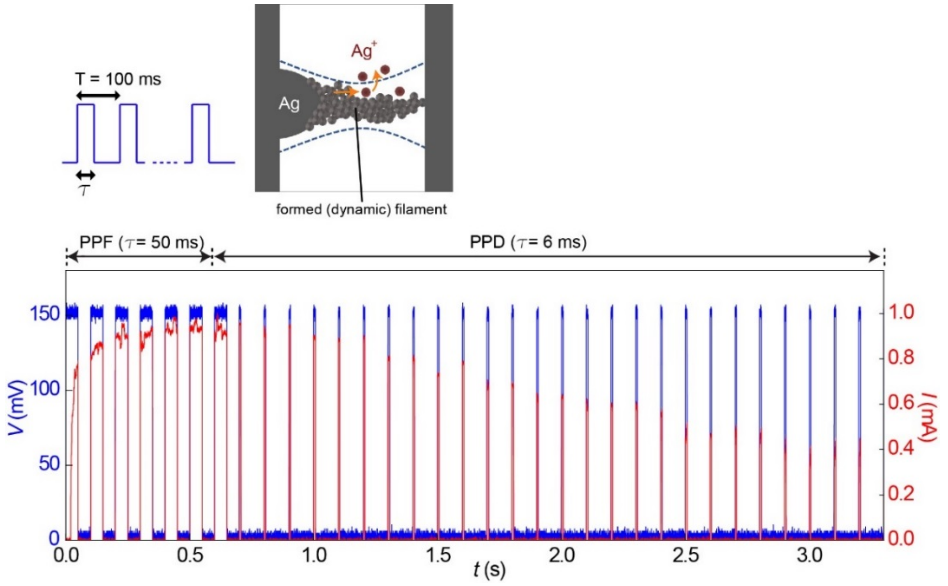

Supplementary Figure 21 | Conduction modulation by pulse width in protein-nanowire synapse made from a planar memristor. (Top panel) Schematic of the input pulse train to an activated protein-nanowire memristor. The pulse frequency (f = 10 Hz) or period (T = 100 ms) is fixed, whereas the pulse width (τ) is modulated. (Bottom panel) At τ=50 ms, the activated nanowire synapse showed increasing conductance (red curve) or paired pulse facilitation (PPF) with increased number of pulses. Reduced pulse width (τ=6 ms) resulted in a decrease in conductance or paired pulse depression (PPD) with increased number of pulses.

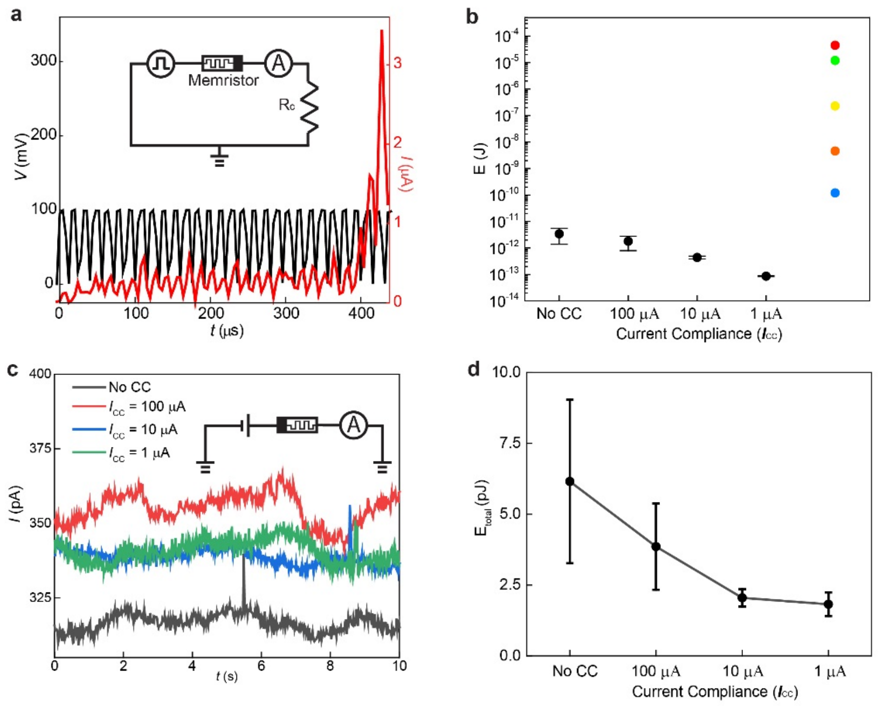

Supplementary Figure 22 | Dynamic power in neuronal spiking. (**a**) Representative (red) integrate-and-fire process in an artificial neuron by using (black) bio-voltage pulses (100 mV, 10 µs width, 15 µs period). Current compliance (I_{CC}) can be added by connecting a compliance resistor (R_C). (**b**) Energy consumption (E) in the spiking event at different I_{CC} in our device, compared to estimated values (color dots) from previously reported neurons.[17-21] The data shows that even without current compliance, the energy cost in our device is still close to two orders of magnitude lower than the lowest reported value. (**c**) To include the energy cost in the incubation period (*i.e.*, leaky current before spiking), the Off currents were measured (the device was reversely biased to prevent turning On). (**d**) The total energy cost (E_{total}) in a spiking event, as the summation of dynamic energy in (**b**) and energy loss through leaky current in (**c**) during the incubation period. The error bars in (**b**) and (**d**) represent the standard deviation (s.d.).

The initiation of an action potential in biological cells is estimated to consume 10^7-10^8 ATP.[22,23] This leads to an estimated 1-10 pJ energy cost (*e.g.*, based on a free energy of ~60 kJ/mol during the hydrolysis of ATP to ADP). Therefore, the energy cost in the bio-voltage neuronal spiking is close to or even below that of a biological cell.

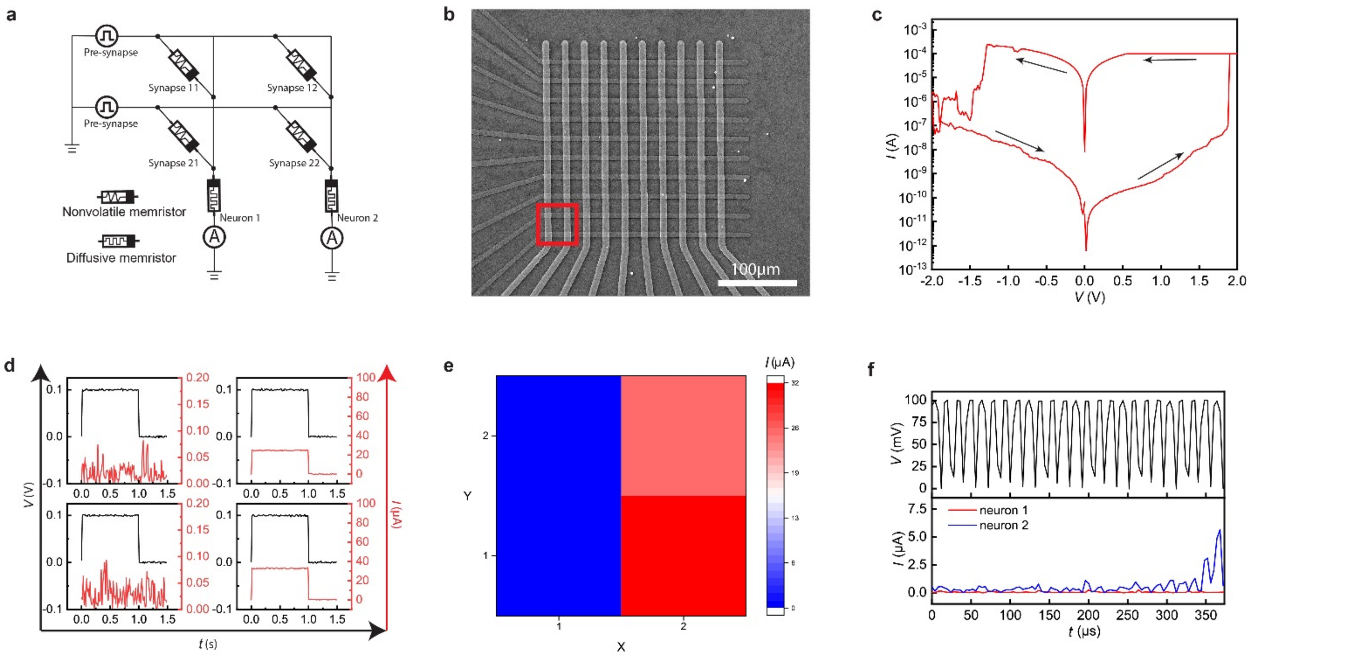

Supplementary Figure 23 | Implementation of bio-voltage neurons in neural network. (**a**) Previous work has demonstrated efficient pattern classification using a fully memristive neural network integrated with both synapses (by nonvolatile memristors) and neurons (by diffusive memristors).[21] Without losing generality, here we schematically represent the network consisting of a 2×2 synapse matrix and 2 neurons (made from bio-voltage diffusive memristors). The pre-synaptic input vector is first computed by the synapse matrix, forming the post-synaptic signal vector that further feeds into the neuron array. The neuron array integrates the post-synaptic vector (or weighted synaptic signals) to form the output vector. (**b**) SEM image of a 10×10 synapse matrix in a crossbar architecture, each synapse being a nonvolatile memristor made of $Ag/SiO_2/Pt$ (150nm/35nm/20nm).[24] We used a 2×2 synapse matrix (red box) from it. (**c**) Characteristic switching *I-V* from a synapse in the crossbar, showing a set voltage ~ 1.8 V and reset voltage ~ -1.5 V. (**d**) The weight/conduction in each pre-trained synapse (read by 100 mV), which is further represented in a color map in (**e**). (**f**) Spiking amplitude of 100 mV (top) was used for post-synaptic readout, which was subsequently integrated by the neurons (owing to the bio-voltage function) for different outputs (bottom). The energy cost in this reading process was substantially reduced by using bio-voltage neurons, as analyzed before (Supplementary Figure 22). If we could replace the high-voltage (*e.g.*, $V_T > 1V$) synapses with bio-voltage (*e.g.*, $V_T < 100$ mV) synapses, the entire unsupervised training/learning[21] could be realized with 100 mV spikes to yield substantial power reduction as well.

Supplementary References

1. Lovley, D. R. & Walker, D. J. F. Geobacter protein nanowires. *Front. Microbiol.* **10**, 2078 (2019).
2. Wang, F. *et al*. Structure of microbial nanowires reveals stacked hemes that transport electrons over micrometers. *Cell* **177**, 361–369 (2019).
3. Filman, D. J. *et al*. Cryo-EM reveals the structural basis of long-range electron transport in a cytochrome-based bacterial nanowire. *Commun. Biol.* **2**, 219 (2019).
4. Puzyrev, Y. S. *et al*. Memristive devices from ZnO nanowire bundles and meshes. *Appl. Phys. Lett*. **111**, 153504 (2017).
5. O'Callaghan, C. *et al*. Collective capacitive and memristive responses in random nanowire networks: Emergence of critical connectivity pathways. *J. Appl. Phys*. **124**, 152118 (2018).
6. Smith, J. A., Meier, D., Woodard, T. L. & Lovley, D. R. Limited role of cytochrome filaments in long-range extracellular electron transfer, in review.
7. Adhikari, R. Y., Malvankar, N. S., Tuominen M. T. & Lovely, D. R. Conductivity of individual Geobacter protein nanowires. *RSC Advances* **6**, 8354-8357 (2016).
8. Wang, Z. *et al*. Threshold switching of Ag or Cu in dielectrics: materials, mechanism, and applications. *Adv. Funct. Mater*. **28**, 1704862 (2018).
9. Hou, H. *et al*. Interfacial activity of amine-functionalized polyhedral oligomeric silsesquioxanes (POSS): a simple strategy to structure liquids. *Angew. Chem*. **131**, 10248-10253 (2019).
10. Song, B., Hu, X., Shui X., Cui, Z. & Wang, Z. A new type of renewable surfactants for enhanced oil recovery: dialkylpolyoxyethylene ether methyl carboxyl betaines. *Colloids and Surfaces A: Physicochem. Eng. Aspects* **489**, 433–440 (2016).
11. Yu, M., Funke, H. H., Falconer, J. L. & Noble, R. D. Gated ion transport through dense carbon nanotube membranes. *J. Am. Chem. Soc.* **132**, 8285-8290 (2010).
12. Cui, Y., Zhong, Z., Wang, D., Wang, W. U. & Lieber, C. M. High performance silicon nanowire field effect transistors. *Nano Lett*. **3**, 149–152 (2003).
13. Fan, Z. *et al*. Wafer-scale assembly of highly ordered semiconductor nanowire arrays by contact printing. *Nano Lett*. **8**, 20-25 (2008).
14. Gao, H. *et al*. Deterministic assembly of three-dimensional suspended nanowire structures. *Nano Lett*. **19**, 5647-5652 (2019).
15. http://www.chinstruments.com/chi400.shtml
16. Feng, J. *et al.* Giant moisture responsiveness of VS_2 ultrathin nanosheets for novel touchless positioning interface. *Adv. Mater*. **24**, 1969-1974 (2012).
17. Adda, C. *et al*. Mott insulators: A large class of materials for Leaky Integrate and Fire (LIF) artificial neuron. *J. Appl. Phys*. **124**, 152124 (2018).
18. Lashkare, S. *et al.* PCMO RRAM for integrate-and-fire neuron in spiking neural networks. *IEEE Electron Device Lett.* **39**, 484-487 (2018).
19. Zhang, X. *et al.* An artificial neuron based on a threshold switching memristor. *IEEE Electron Device Lett*. **39** 308-311 (2017).
20. Stoliar, P. *et al*. A leaky-integrate-and-fire neuron analog realized with a Mott insulator. *Adv. Funct. Mater*. **27**, 1604740 (2017).
21. Wang, Z. *et al.* Fully memristive neural networks for pattern classification with unsupervised learning. *Nat. Electron.* **1**, 137-145 (2018).
22. Hasenstaub, A., Otte, S., Callaway, E. & Sejnowski, T. J. Metabolic cost as a unifying principle governing neuronal biophysics. *Proc. Natl. Acad. Sci. USA* **107**, 12329-12334 (2010).
23. Sengupta, B., Stemmler, M., Laughlin, S. B. & Niven, J. E. Action potential energy efficiency varies among neuron types in vertebrates and invertebrates. *PLoS Comput. Biol*. **6**, e1000840 (2010).
24. You, B. K. *et al.* Reliable memristive switching memory devices enabled by densely packed silver nanocone arrays as electric-field concentrators. *ACS Nano* **10**, 9478-9488 (2016).
